# Referees 

**DOI:** 10.1111/gbb.12878

**Published:** 2023-12-20

Alexander, Matthew.

Anokhin, Andrey.

Atkinson, Nigel.

Bachtell, Ryan.

Bajo, Michal.

Bales, Karen.

Barrot, Michel.

Bast, Tobias.

Beckstead, Michael.

Belleau, Emily.

Besheer, Joyce.

Biernacka, Joanna.

Blundell, Jacqueline.

Bordt, Evan.

Bosch, Oliver.

Brown, Richard.

Bryant, Camron.

Candlish, Michael.

Cavanaugh, Daniel.

Cheatwood, Joseph.

Chester, Julia.

Chowdhury, Budhaditya.

Clarke, Gerard.

Clayton, David.

Colwell, Christopher.

Cook, Melloni.

Couto, Antoine.

Cushing, Bruce.

De Fraga,

Deak, Joseph.

Diaz, Heijtz,

Dijkstra, Peter.

Ehlers, Cindy.

Fendt, Markus.

Ferguson, David.

Fuchsova, Beata.

Furtjes, Anna.

Geiger, Madeleine.

Geleoc, Gwenaelle.

Gizer, Ian.

Glover, Elizabeth.

Greeson, Ben.

Grinevich, Valery.

Gu, Bin.

Hammack, Sayamwong.

Hatoum, Alexander.

Hauber, Mark.

Hendricks, Audrey.

Hill, Shirley.

Hopf, Frederic.

Johnson, Emma.

Johnson, Michael.

Jurek, Benjamin.

Kenkel, William.

Kiraly, Drew.

Konopka, Genevieve.

Kopec, Ashley.

Kuhn, Donald.

Kumar, Vivek.

Larue, Lionel.

LaSarge, Candi.

Lipina, Tatiana.

Logan, Ryan.

Lopez, Sofia.

Lord, Kathryn.

Lucas, Guilherme.

Luciano, Michelle.

Lugo, Joaquin.

Mayfield, Roy.

McDermott, Brian.

Mello, Claudio.

Menon, Rohit.

Miller, Alex.

Miranda‐Barrientos, Jorge.

Mosienko, Valentina.

Moy, Sherly.

Mulligan, Megan.

Murlanova, Kateryna.

Ozburn, Angela.

Pathak, Gita.

Patten, Shunmoogum.

Paul, Sarah.

Pawluski, Jodi.

Pignataro, Giuseppe.

Pletnikov, Mikhail.

Ploski, Jonathan.

Ponomarev, Igor.

Powell, Susan.

Pryce, Christopher.

Raber, Jacob.

Ragozzino, Michael.

Randall, Patrick.

Rasband, Matthew.

Redei, Eva.

Riley, Brien.

Roman, Gregg.

Schmidtner, Anna.

Schreihofer, Derek.

Serafini, Gianluca.

Sherva, Richard.

Siegel, Jessica.

Snyder, Brina.

Sokoloff, Greta.

Sorenson, Michael.

Stitzel, Jerry.

Tabakoff, Boris.

Terman, Jonathan.

Thomas, Nathaniel.

Thorogood, Rose.

Wall, Tamara.

Waterland, Robert.

Wedow, Robbee.

Willadsen, Maria.

Williams, Aislinn.

Wray, Naomi.

Yan, Hua.

Yoshino, Yuta.

Young, Andrew.

